# Silencing cyclophilin A improves insulin secretion, reduces cell apoptosis, and alleviates inflammation as well as oxidant stress in high glucose-induced pancreatic β-cells via MAPK/NF-kb signaling pathway

**DOI:** 10.1080/21655979.2020.1823729

**Published:** 2020-09-24

**Authors:** Tangying Li, Huibiao Quan, Huachuan Zhang, Leweihua Lin, Qianying Ou, Kaining Chen

**Affiliations:** a Department of Health Care Centre, Hainan General Hospital, Haikou, Hainan, China; b Department of Endocrinology, Hainan General Hospital, Haikou, Hainan, China; c Department of Endocrinology Laboratory, Hainan General Hospital, Haikou, Hainan, China

**Keywords:** Cyclophilin A, apoptosis, inflammation, oxidant stress, pancreatic β-cells, MAPK/NF-kb signaling pathway

## Abstract

Cyclophilin A is increased in the plasm of diabetic patients, while its effects on high glucose (HG)-stimulated pancreatic β-cells are still pending. The aim of this research is to investigate the effects of cyclophilin A inhibition on HG-challenged pancreatic β-cells. For investigating the effects of cyclophilin A decrease on HG-induced pancreatic β-cells, the cells were separated into normal glucose (NG), Mannitol, HG, HG + shRNA-NC, and HG + shRNA-Cyclophilin A-1 groups. The protein and mRNA expression were detected via Western blot and qRT-PCR. CCK-8 assay and flow cytometry were employed for assessing cell viability and apoptosis. The levels of oxidative stress, inflammation, and insulin secretion were detected by corresponding kits. The cyclophilin A was higher in HG group. Knockdown of cyclophilin A was able to increase insulin secretion, decrease cell apoptosis, and alleviate inflammation as well as oxidant stress in HG-treated pancreatic β-cells via MAPK/NF-kb pathway. Taken together, Cyclophilin A, highly expressed in pancreatic β-cells induced by HG, is a promising therapeutic target for diabetes. Knockdown of cyclophilin A has protective effects against HG-challenged pancreatic β-cells via regulation of MAPK/NF-kb pathway. The findings in this study provided a new strategy for diabetic treatment and paved the way for future researches on diabetes treatment.

## Graphical Abstract

**Figure:**
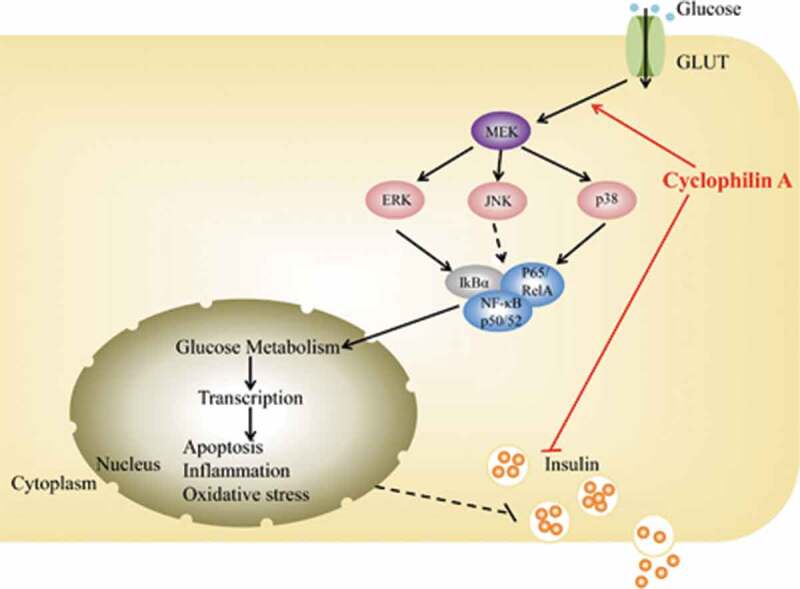

## Introduction

Diabetes mellitus has been a major public health problem worldwide [1,2]. The number of the patients with diabetes is on the surge and it is projected that the diabetes patients will reach about 600 million twenty years later [3,4]. Diabetes can result in organ dysfunctions and even a series of complications, such as endothelial dysfunction, diabetic retinopathy, nephropathy, and so forth [5–9]. Therefore, it is urgent to explore the mechanism, occurrence, and development of diabetes and reveal the possible effective targets or agents for therapy of diabetes.

Type 1 diabetes (T1D) and type 2 diabetes (T2D) are the major forms of diabetes [10,11]. T1D is attributed to destruction of the pancreatic β cells or inhibition of glucose uptake [12,13]. T2D is due to pancreatic β cells function decline together with insulin resistance [14]. Ninety-five percent of diabetic patients suffered from T2D [15,16]. Hence, searching for the effective therapeutic target in T2D is beneficial for the majority of diabetic patients.

Cyclophilin A, a highly conserved protein, mainly exists in cytoplasm, regulating protein folding, transport, and cytokine secretion [17]. Besides, cyclophilin A acts as an crucial player in multiple human diseases such as atherosclerosis, hepatitis virus, cancer, cardiac diseases, and so forth [18].

The secretion of cyclophilin A can be provoked by some pathological conditions including oxidative stress, hypoxia, and inflammation [19–21]. Oxidative stress and inflammation are the major causes of diabetes. Cyclophilin A level in plasma is reported to be elevated in patients suffering from T2D, indicating the occurrence of vascular disease [22]. Diabetes is characterized by high glucose (HG) level. Cyclophilin A secretion is increased by HG and oxidant stress in diabetic nephropathy [23]. Moreover, cyclophilin A expression is also increased in HG-induced macrophages. However, the exact function of cyclophilin A in glucose-treated pancreatic β-cells remains unknown.

In the current research, our objective was to explore the possible impacts of cyclophilin A on pancreatic β-cells with HG induction and investigate its underlying mechanism. Our findings revealed that inhibition of cyclophilin A promoted insulin secretion, attenuated cell apoptosis rate, and alleviated inflammation as well as oxidant stress in pancreatic β-cells stimulated by HG. Hence, cyclophilin A may be a potential target for diabetes treatment.

## Material and methods

### Cell culture and treatment

The INS-1 cell line was procured from Xinzhou Biotechnology Company (Shanghai, China). The cells (1 × 10^5^ cells/well) were grown in a 96-pore plate in RPMI 1640 medium with FBS (10%), β-mercaptoethanol (50 μM), streptomycin 100 (μg/ml) as well as penicillin (100 U/ml). The cells were cultivated in an incubator of 5% CO_2_ [24]. For evaluating the levels of cyclophilin A, the cells were divided into three different groups. In normal glucose (NG) group, 5 mM glucose (normal glucose concentration) was used; in Mannitol group, 33 mM Mannitol was utilized and in HG group, 33 mM glucose was applied. Cells from each group were incubated for 48 h with or without HG treatment [25]. The NG served as control and Mannitol group as an osmotic control. For screening the efficacy of plasmid downregulating cyclophilin A expression, the cells were designed into HG, HG + shRNA-NC, HG + shRNA-Cyclophilin A-1, and HG + shRNA-Cyclophilin A-2 groups. For evaluating the effects of cyclophilin A depletion on HG-treated pancreatic β-cells, the cells were designed into NG, MG, HG, HG+shRNA-NC, and HG+shRNA-cyclophilin A groups.

### Cell transfection

The cells were seeded in a 6-well plate in RPMI 1640 medium (Sigma, Darmstadt, Germany). The cells were transfected with shRNA-NC, shRNA-Cyclophilin A-1 and shRNA-Cyclophilin A-2 in accordance with the manufacturer’s protocols. shRNA-NC, shRNA-Cyclophilin A-1 (5′-GCCATCGAGAAGGTTGGATCA-32032), and shRNA-Cyclophilin A-2 (5′-GGTGCAAACACGAACGGATCT-3′) mentioned above were purchased from Shanghai GenePharma company.

### Western blot

The cells of different groups were lysed to collect the total proteins, as previously described [26]. The Bradford assay was performed to evaluate the protein concentrations. The proteins were separated with application of 10% SDS-polyacrylamide gel electrophoresis and next transferred onto PVDF membranes (Millipore, USA). Five percent skim milk was employed to block the membranes and subsequently the membranes were subjected to culture with primary antibodies against Cyclophilin A (#ab41684, Abcam), bcl2 (#ab196495, Abcam), Bax (#ab32503, Abcam), cleaved caspase3 (#ab49822, Abcam), caspase3 (#ab13847, Abcam), Cleaved Caspase-9 (#9509, Cell Signaling Technology), caspase 9 (#ab202068, Abcam), JNK (#ab199380, Abcam), P-JNK (#ab47337, Abcam), ERK (#ab32537, Abcam), P-ERK (#ab131438, Abcam), P38 (#ab170099, Abcam), P-P38 (#ab4822, Abcam), p-NF-KB P65 (#ab28856, Abcam), p-IKBα (#ab24783, Abcam), NF-KB P65 (#ab16502, Abcam), and IKBα (#ab32518, Abcam). Thereafter, the membranes were co-incubated with the horseradish peroxidase-conjugated secondary antibody (Cell Signal Technology). The enhanced chemiluminescence kit (Thermo Fisher Scientific, Waltham, Massachusetts, USA) was utilized to detect the protein blots.

### Detection by quantitative real-time PCR (qRT-PCR)

The total RNAs were harvested through TRIzol reagent (Invitrogen, Carlsbad, CA, USA). Synthesis of cDNA was performed utilizing the PrimeScript RT Master Mix kit (Takara Bio, Inc., Otsu, Japan). ABI7900 Real-time PCR System (Applied Biosystems, Foster City, USA) was applied for qRT-PCR assay. The conditions of PCR were shown as follows: 95°C for 6 min, with 40 cycles of 95°C for 10 s, 60°C for 25 s, 70°C for 30s. The primers for cyclophilin A were: Forward 5′-CAAGGTCCCAAAGACAGCAGA-3′ and Reverse 5′-AAGATGCCAGGACCCGTATGC-3′. 2^–ΔΔCT^ method was applied for calculation of the relative expression [27].

### Cell counting kit-8 (CCK-8) assay

The cells (1 × 10^5^) were placed on a 96-well culture dish. After treatment in the corresponding groups, the cells were incubated with CCK-8 agent (DOJINDO, Laboratories, Kumamoto, Japan) for 1 h at 37°C. Next, cell viability was determined through measuring the absorbance at 450 nm [28]. For this procedure, the microplate reader (Bio-Tek, Winooski, USA) was used.

### Assessment of insulin secretion

The cells of different groups, after sonication in acid methanol, were centrifuged for 8 min at 4°C for the supernatant collection. Insulin ELISA kit (Thermo Fisher Scientific) was utilized for detecting the insulin levels, as per the manufacturer’s guidance.

### Detection of inflammation factors and oxidant stress level

IL-1β, TNF-a, and IL-18 as inflammatory indicators and ROS, MPO, and SOD as indicators of oxidant stress were evaluated herein. The cells of different groups were lysed and centrifuged for collecting the supernatants. The levels of inflammation and oxidant stress were detected via the corresponding kits, following the manufacturer’s guideline.

### Assessment of cell apoptosis

Flow cytometry (BD Biosciences, New Jersey, USA) was applied to detect the cell apoptosis in different groups as previously described [29]. Briefly, the cells of different groups were harvested and rinsed with PBS. Then the cells were incubated with annexin V-FITC (5 μL) and PI (5 μL) for another 20 min away from the light. After staining, the cell apoptosis was evaluated by flow cytometry.

### Statistical analysis

All the experiments were repeated thrice and the obtained results were presented as mean ± SD. The GraphPad Prism 5.0 software (GraphPad Software, Inc, La Jolla, CA) was applied for data analysis. As for differences, one-way or two-way ANOVA followed by Tukey’s multiple comparison tests was conducted.

## Results

### The cyclophilin A level was increased by HG in pancreatic β-cells

**Figure 1.**
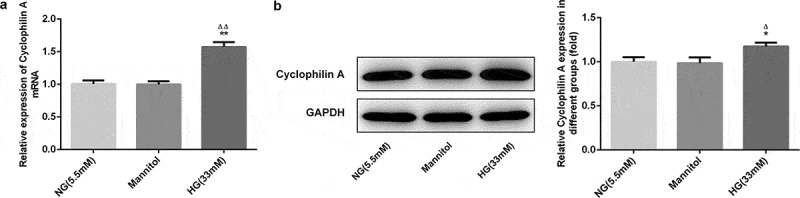
The cyclophilin A level evaluated by qRT-PCR (a) and Western blot (b) in pancreatic β-cells.

The cyclophilin A level in NG group was nearly the same to that in Mannitol group, indicating that the cyclophilin A level was not influenced by Mannitol (Figure 1). Compared with NG group and Mannitol group, the cyclophilin A level was higher in HG group, demonstrating that the secretion of cyclophilin A was increased by HG and its inhibition may protect against HG-caused injury in pancreatic β-cells.

*P < 0.05 and **P < 0.01 vs. NG group, ^Δ^P < 0.05 and ^ΔΔ^P < 0.01 vs. Mannitol group.

### Knockdown of cyclophilin A increased the cell viability of HG-stimulated pancreatic β-cells

**Figure 2.**
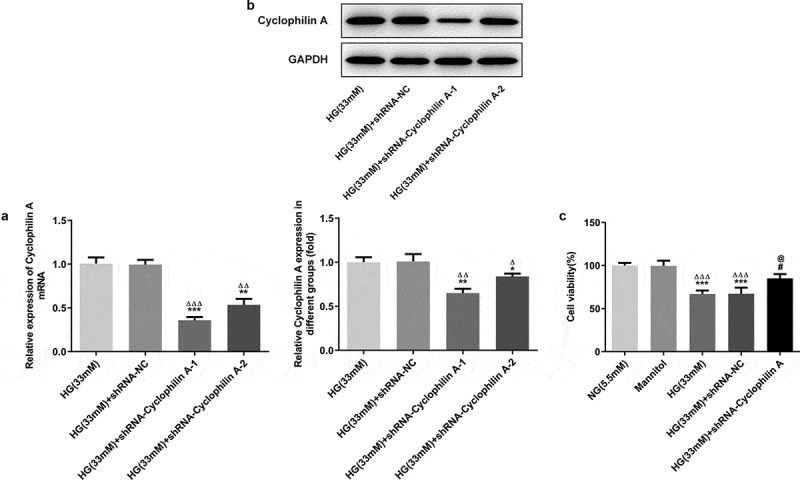
The effects of cyclophilin A silencing on the cell viability in HG-treated pancreatic β-cells.

As examined by qRT-PCR, the cyclophilin A level is not alerted by shRNA-NC but downregulated efficiently by transfection of shRNA-Cyclophilin A-1 (Figure 2(a and b)). No significant changes of cell viability between NG group and Mannitol group were found, suggesting that high concentration of Mannitol had no effect on cell viability. The cell viability was reduced significantly in HG group in contrast to NG group, indicating that HG resulted in cell injury (Figure 2(c)). After knockdown of cyclophilin A, the cell viability of pancreatic β-cells in HG group was increased, confirming that knockdown of cyclophilin A have promoting effect on cell viability in pancreatic β-cells treated by HG.

The cyclophilin A level evaluated by PCR (a) and Western blot (b) in the study groups. The cell viability level in the study groups (c).*P < 0.05 and **P < 0.01 and ***P < 0.01 vs. HG (33 mM) group, ^Δ^P < 0.05 and ^ΔΔ^P < 0.01 and ^ΔΔΔ^P < 0.01 vs. HG (33 mM) +shRNA-NC group (A and B); ***P < 0.001 vs. NG group, ^ΔΔΔ^P < 0.001 vs. Mannitol group, ^#^P < 0.05 vs. HG (33 mM) group, ^@^P < 0.05 vs. HG (33 mM) + shRNA-NC group (C)

### Knockdown of cyclophilin A increased insulin secretion in HG-damaged pancreatic β-cells

**Figure 3.**
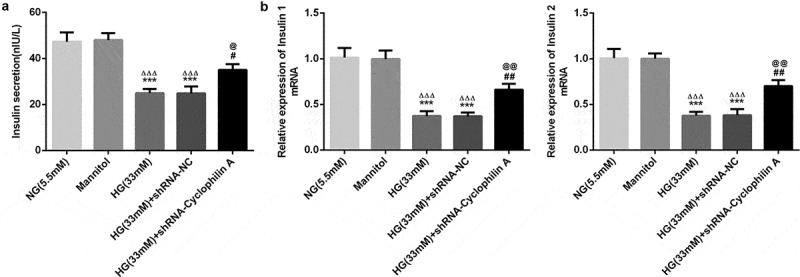
The effects of cyclophilin A silence on insulin secretion in pancreatic β-cells exposed to HG.

As shown in Figure 3(a and b), the insulin secretion was not affected by either Mannitol or shRNA-NC. The insulin secretion was inhibited obviously by HG in contrast to NG group. Moreover, the insulin secretion decreased by HG induction was increased after cyclophilin A was knocked down. The results demonstrated that knockdown of cyclophilin A was able to increase the HG-reduced insulin content.

Insulin secretion level evaluated by insulin ELISA kit (a) and PCR (b) in the study groups.***P < 0.001 vs. NG group, ^ΔΔΔ^P < 0.001 vs. Mannitol group, ^#^P < 0.05 and ^##^P < 0.01 vs. HG (33 mM) group, ^@^P < 0.05 and ^@@^P < 0.01 vs. HG (33 mM) + shRNA-NC group.

### Knockdown of cyclophilin A alleviated inflammatory response and oxidative stress in pancreatic β-cells under exposure to HG

**Figure 4.**
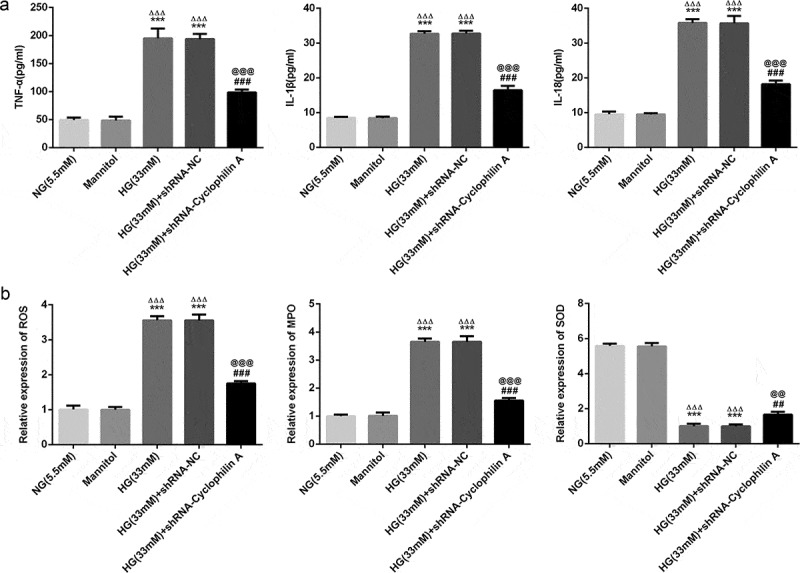
The effects of cyclophilin A decrease on inflammation and oxidative stress in HG-stimulated pancreatic β-cells.

IL-18, TNF-a, and IL-1β, three indicators of inflammation, were evaluated herein. As shown by Figure 4(a), the inflammation level was not alerted by Mannitol and shRNA-NC. But there was a significant increase in inflammation of HG group compared with NG group, indicating that HG increased the inflammation level. Whereas the HG-increased inflammation was decreased by knockdown of cyclophilin A, confirming that knockdown of cyclophilin A had inhibitory effects on HG-promoted inflammation in pancreatic β-cells. After that, SOD, ROS, and MPO as indicators of oxidative stress were evaluated (Figure 4(b)). Mannitol and shRNA-NC had no effects on oxidative stress level. Furthermore, the levels of ROS and MPO were increased and that of SOD was decreased by HG. The effects of HG on these indicators were reversed by knockdown of cyclophilin A. Together, all the findings uncovered that knockdown of cyclophilin A had suppressive effects on levels of inflammation and oxidative stress in HG-stimulated pancreatic β-cells.

The inflammation level (a) and oxidative stress level (b) in the study groups.***P < 0.001 vs. NG group, ^ΔΔΔ^P < 0.001 vs. Mannitol group, ^##^P < 0.01 and ^###^P < 0.001 vs. HG (33 mM) group, ^@@^P < 0.01 and ^@@@^P < 0.001 vs. HG (33 mM) + shRNA-NC group.

### Knockdown of cyclophilin A reduced the cell apoptosis in HG group

**Figure 5.**
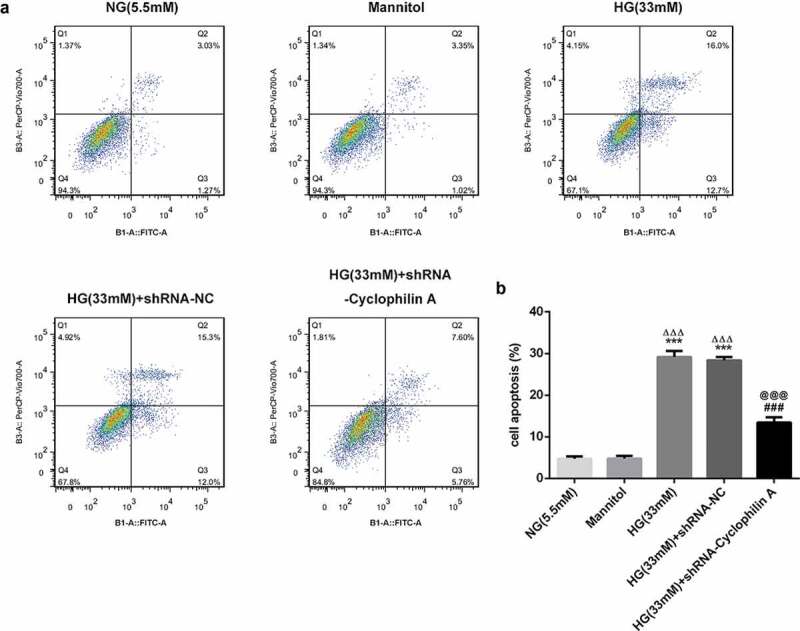
The effects of cyclophilin A silencing on cell apoptosis in HG-treated pancreatic β-cells.

**Figure 6.**
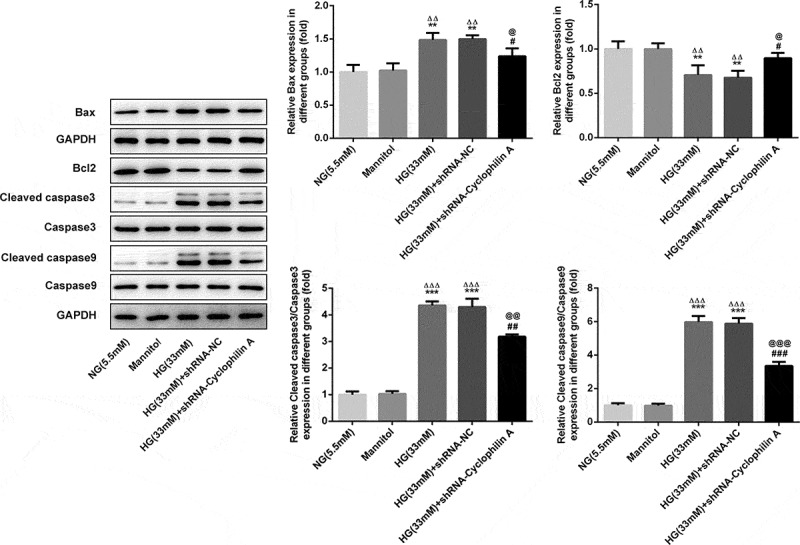
The effects of cyclophilin A decrease on apoptosis-related proteins in HG-induced pancreatic β-cells.

As estimated by flow cytometry, the apoptosis was not changed by Mannitol and shRNA-NC (Figure 5). The cell apoptosis was increased after HG exposure and this effect was restrained by silencing of cyclophilin A, suggesting the attenuative effect of cyclophilin A knockdown on cell apoptosis. The bcl2, bax, cleaved caspase3 and cleaved caspase 9 as apoptosis-related proteins were also evaluated. As seen in Figure 6, the Mannitol and shRNA-NC exerted no effects on expression levels of apoptosis-related proteins. The levels of bcl-2 as an anti-apoptosis protein were decreased and levels of cleaved caspase 3/9 as pro-apoptosis proteins were increased in HG-induced pancreatic β-cells. This result consisted with the finding of flow cytometry, confirming that HG had promoting effects on cell apoptosis. The effects of HG on apoptosis-related proteins were further abrogated by knockdown of cyclophilin A. All the findings confirmed that knockdown of cyclophilin A was able to lessen cell apoptosis activated by HG.

The cell apoptosis level in the study groups (a and b).***P < 0.001 vs. NG group, ^ΔΔΔ^P < 0.001 vs. Mannitol group, ^###^P < 0.001 vs. HG (33 mM) group, ^@@@^P < 0.001 vs. HG (33 mM) + shRNA-NC group.

The levels of apoptosis-related proteins in the study groups.**P < 0.01 and ***P < 0.01 vs. NG group, ^ΔΔ^P < 0.01 and ^ΔΔΔ^P < 0.001 vs. Mannitol group, ^#^P < 0.05, ^##^P < 0.01, and ^###^P < 0.001 vs. HG (33 mM) group, ^@^P < 0.05, ^@@^P < 0.01 and ^@@@^P < 0.001 vs. HG (33 mM) + shRNA-NC group.

### The effects of knockdown of cyclophilin A on proteins of MAPK/NF-kb signaling pathway

**Figure 7.**
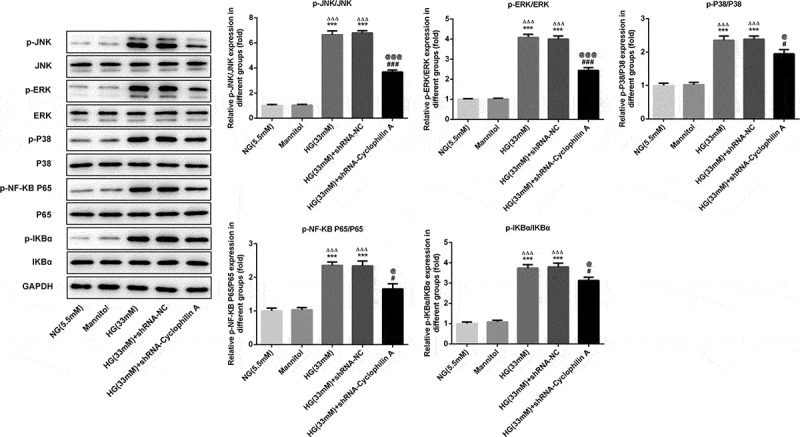
The effects of cyclophilin A downregulation on protein expressions of MAPK/NF-kb signaling pathway in HG-treated pancreatic β-cells.

Cyclophilin A is reported to involve in inflammation by activating MAPK/NF-kb signaling [30]. The expression levels of MAPK/NF-kb pathway proteins were also evaluated. As shown in Figure 7, proteins involved in MAPK/NF-kb pathway were unaffected with the treatment of Mannitol and shRNA-NC. The p-JNK, p-ERK, p-P38, p-NF-KB P65, and p-IKBα levels were all increased by HG treatment and these phenomena were further inhibited by knockdown of cyclophilin A. The results demonstrated that protective effects of cyclophilin A knockdown in HG-damaged pancreatic β-cells was realized via inactivating MAPK/NF-kb signaling.

***P < 0.01 vs. NG group, ^ΔΔΔ^P < 0.001 vs. Mannitol group, ^#^P < 0.05 and ^###^P < 0.001 vs. HG group, ^@^P < 0.05 and ^@@@^P < 0.001 vs. HG (33 mM) + shRNA-NC group.

## Discussion

Nowadays, diabetes mellitus remains to be a major health killer and there are no effective therapies for its treatment [31,32]. The general strategies are glucose control and alimentary control, incapable of radical treatment [33,34]. Finding the endogenous targets that exert substantial roles in the occurrence and development of diabetes mellitus may open up new direction for radical treatment of diabetes mellitus.

Cyclophilin A ubiquitously expressed in varieties of cells has been confirmed as a vital function protein that contributes to diabetic nephropathy, with high level in plasm of patients with diabetes [22,23]. We speculated that cyclophilin A might act as a pivotal role in pancreatic β-cells exposed to HG. Insulin insufficiency resulted from pancreatic β-cell dysfunction is the essential pathogenic factor in diabetes. Based on the theory above, in this research, we first explored the influences of cyclophilin A on HG-treated pancreatic β-cells. The results unmasked that knockdown of cyclophilin A improved insulin secretion, reduced cell apoptosis, and alleviated inflammation as well as oxidant stress in pancreatic β-cells under exposure to HG via repressing MAPK/NF-kb pathway.

HG-challenged pancreatic β-cells are the common diabetic cell model, as reported by many researches [35–37]. We next assessed the level of cyclophilin A, which unveiled that the level of cyclophilin A was higher in diabetic cell model. We speculated that knockdown of cyclophilin A exerted protective effects in pancreatic β-cells exposed to HG. HG has pro-apoptotic property in pancreatic β-cells [38–40]. Consistent with previous researches, the cell viability was decreased, and cell apoptosis was promoted in HG group, suggesting that the diabetic cell model was established successfully. The pro-apoptosis protein level was increased, and antiapoptosis protein level was decreased by HG, which was reversed by introduction of cyclophilin A knockdown plasmids. In a word, knockdown of cyclophilin A increased the cell viability and decreased the cell apoptosis affected by HG. Pancreatic β-cell apoptosis inevitably causes insufficient insulin, which is a vital factor in the initiation and progress of diabetes. We further evaluated the insulin content as the further evidence. The insulin content was decreased by HG treatment, which was possibly associated with the high cell apoptosis rate in HG-induced pancreatic β cells. Previous study reported that insulin secretion was reduced under the environment of HG [41]. Moreover, decreased blood glucose alleviated apoptosis of islet β cells and increased insulin secretion [42]. In present study, knockdown of cyclophilin A increased insulin secretion in HG-damaged pancreatic β-cells. Thus, knockdown of cyclophilin A may be a promising strategy for radical treatment of diabetes. Inflammation and oxidative stress are two major factors contributing to diabetes-induced injuries [43,44]. And inhibition of inflammation and oxidative stress develop protective effects in diabetes [45–48]. Cyclophilin A is confirmed to be secreted by cells under the stimulation of inflammation and oxidative stress [49]. In addition, cyclophilin A serves as the major downstream factor of oxidative stress and inflammatory cytokines [50–53]. In the current work, the levels of inflammation and oxidative stress stimulated by HG was restricted by knockdown of cyclophilin A, portending cyclophilin A as a considerable therapeutic target for diabetes.

The signaling pathway underlying cyclophilin A in diabetes was also explored primarily. MAPK/NF-kb signaling pathway is an inflammation-related pathway and plays a pivotal role in inflammation [54,55]. The MAPK/NF-kb is activated when cyclophilin A partakes in inflammation [56]. In this paper, the MAPK/NF-kb pathway was activated under HG exposure, which may be the mechanism accounting for high inflammation level in HG group. Furthermore, after knockdown of cyclophilin A, the proteins of MAPK/NF-kb pathway induced by HG was markedly down-regulated, hinting that MAPK/NF-kb signaling was hampered by knockdown of cyclophilin A and the protective effects of cyclophilin A silence may be partly attributed to inactivation of the MAPK/NF-kb signaling pathway.

In summary, cyclophilin A was an important function protein in diabetes and its knockdown exerted protective effects on pancreatic β-cells against HG via inhibiting MAPK/NF-kb signaling. Hence, cyclophilin A may be a potential target for diabetes treatment. Our study provided new insights into understanding the pathogenesis of diabetes and presented novel therapeutic strategies for its treatment. Additionally, further experiments will be performed to investigate the specific mechanism underlying the role of cyclophilin A in diabetes progression.

## Conclusion

In this research, the results uncovered that knockdown of cyclophilin A possessed protective effects in HG-challenged pancreatic β-cells via MAPK/NF-kb pathway, offering a new strategy for treatment of diabetic. The role of cyclophilin A knockdown in vivo still needs further study. In addition, MAPK/NF-kb signaling may be not the unique signaling implicated in the role of cyclophilin A. In-depth researches are still needed to illuminate the role of cyclophilin A in diabetes.

## Highlights

- The cyclophilin A level was increased by HG in pancreatic β-cells
- Knockdown of cyclophilin A increased the cell viability and reduced the cell apoptosis in HG group
- Knockdown of cyclophilin A increased insulin secretion in HG-damaged pancreatic β-cells
- Knockdown of cyclophilin A suppressed inflammation and oxidative stress in pancreatic β-cells under exposure to HG
